# Biomechanical components of the plant–insect herbivore arms race: a model test in leaf-cutter ants

**DOI:** 10.1098/rsif.2025.0091

**Published:** 2025-09-10

**Authors:** Olivia K. Walthaus, Dilanka I. Deegala, Raphael Delattre, David Labonte

**Affiliations:** ^1^ Department of Bioengineering, Imperial College London, London, UK

**Keywords:** cutting, insect herbivory, plant mechanical defences, fracture

## Abstract

Insects and plants have been locked in an evolutionary arms race spanning 350 million years. Insects evolved specialized tools to cut into plant tissue, and plants, to counter these attacks, developed diverse defence strategies. Much previous worked has focused on chemical defences. How can plants vary their mechanical properties to deter herbivores, and how can insects respond? We test a simple mechanical model that relates the force required to cut thin, leaf-like tissues to their mechanical properties and the geometry of the cutting tool. To remove the confounding effects of tool shape across size, we use leaf-cutter ant mandibles as a model system. Cutting forces were measured for pristine and worn mandibles that vary by one order of magnitude in size, using a custom-built fibre-optic set-up and homogeneous pseudoleaves as well as a set of plant tissues as model substrates. The results substantially support the model, enabling quantitative predictions. Fracture toughness is identified as a key mechanical defence trait for plants, cutting edge radius as the critical geometric property of the insect mandible and cutting edge wear consequently emerges as a key modulator of cutting forces, elevating it up to fivefold above a physical minimum. Thus, plants may be served by implementing strategies that maximize wear, whereas insects should seek to minimize it.

## 1. Introduction

Insects and plants have co-existed for over 350 million years [1], and a variety of relationships have evolved between them. Some, such as pollination, are mutually beneficial [2]; others, however, are deleterious. Perhaps the most important interaction is the consumption of plants by herbivorous insects [3]. The ecological impact of insect herbivores is vast: they directly affect plant selection, fitness and geographic range, and can act as biological control of invasive plant species [4–9]. But they also have devastating economic effects: although only about 1% of insect species are classified as pests [10], they consume a staggering 13% of crops globally [11] and cause $17.7 billion of damage per year in the US alone [12]. Despite the apparent success of insect herbivores, plant feeding is no trivial matter: it is mechanically challenging and metabolically costly [13–15]. In order to overcome mechanical demands, large head muscles and specialized mouthparts evolved in insects [14,16–19], and in order to keep these demands large, deterrence strategies evolved in plants. Chemical defences, such as the inclusion of flavonoids, terpenoids, tannins and alkaloids, or of secondary metabolites that are poisonous, repellent or even trap herbivorous insects, are well-studied [1,20,21]. However, because the efficacy of chemical defences is dictated by predator physiology—and because they are often only triggered through insect attacks—it is increasingly held that mechanical adaptations are a key line of first defence [13,22–24]. Despite this broadening in perspective, it is not always clear which mechanical plant traits are most effective at deterring herbivores [23,25], nor exactly how they should be measured—some have even suggested that the dominant focus on chemical defences may be partially owed to the absence of reliable biomechanical assays for the determination of key mechanical properties [24]. Morphological features such as waxy cuticles and thorns are examples of specialized and effective mechanical deterrents [1], but perhaps the most relevant mechanical traits are those that increase the physical barrier to plant fracture itself, such as leaf toughening through cell wall thickening or lignification [1,20,26]. No matter the size of the herbivore or the specific plant in question, all herbivory involves a mechanical interaction between mouthpart and plant tissue; and this shared element imposes a number of shared physical constraints. A necessary condition for plant feeding in biting–chewing insects is that the bite force—the maximum force an insect can generate—exceeds the cutting force—the force required to initiate and propagate a cut through the plant tissue with the mandible [13,23,27,28]. The magnitude of the bite force is determined by the morphology and physiology of the musculoskeletal bite apparatus (e.g. [16,29–34]); it is thus independent of the material being cut. The cutting force, in turn, depends both on the material properties of the substrate and the geometry of the ‘tool’ that is used to make the cut [28,35–38]. How do these factors come together to determine the total mechanical effort?

### 1.1. A biomechanical analysis of thin leaf cutting

Cutting is a fracture process—it involves the creation of new surface area, so that cut progression incurs an energetic cost $dU_{a}$. This cost must be supplied by doing work with an external load, $dU_{\text{ext}}=dU_{a}$. From this simple energy balance, it follows that the force F required to cut a substrate of thickness t is bound from below by [36,39–41]


(1.1) $$F\geq G_{c}t,$$

where $G_{c}$ is the critical surface energy per unit crack area. Notably absent from this expression are terms that reflect the geometric properties of the tool used to make the cut—the model predicts the same cutting force for a sharp razor blade or a blunt teaspoon. This prediction arises because the simple energy balance $dU_{\text{ext}}=dU_{a}$ neglects additional energy sinks, arising, e.g. from elastic bending, plasticity or friction [36]. In thin sheet cutting, contributions from bending are likely negligible, but the cutting tool can demand additional energetic costs if it is insufficiently sharp. Assuming that cutting tool shape can be parametrized with one characteristic length scale, R, dimensional arguments suggest that the energetic cost associated with tool bluntness takes the form $dU_{\text{loss}}\approx C\sigma_{c}\text{Rtdx}$, where C is a dimensionless constant of order unity, and $\sigma_{c}$ is a characteristic stress [28,36,38,41]. It follows that


(1.2) $$F_{f}=G_{c}t+C\sigma_{c}Rt.$$

The first term represents the unavoidable energetic cost arising from fracture alone; the second term accounts for additional costs incurred by interactions between the mandible and plant material close to the crack tip. The importance of plant properties and mandible geometry in determining the cutting force thus depends on the relative magnitude of these two terms. An intuitive way to assess their relative contributions is to normalize equation (1.2) with the lower bound cutting force, $G_{c}t$, to obtain the dimensionless form [28]


(1.3) $$\frac{F_{f}}{G_{c}t}=1+C\frac{\sigma_{c}R}{G_{c}}.$$

This writing reveals that the relative importance of plant properties versus tool geometry is governed to first order by the ratio of two length scales: the characteristic mandible dimension, R, and the material length scale, $G_{c}\sigma_{c}^{-1}$. For $R>>G_{c}\sigma_{c}^{-1}$, the cutting force is dominated by dissipative processes near the crack tip and depends on mandible geometry; and for $R<<G_{c}\sigma_{c}^{-1}$, it is close to the physical minimum and depends solely on plant leaf properties—a cutting tool for which $F_{f}/(G_{c}t)\approx 1$ may thus be considered ideally sharp [28,38,41,42]. Previous work provided preliminary evidence in support of this model for insect mandibles [28], but it remained restricted to measurements with one model substrate—more robust and targeted testing is thus required. In this work, we take this model as a starting point and seek to validate it further by systematically testing two immediate predictions that follow from it:

— Cutting force should be proportional to leaf thickness and toughness for $R<<G_{c}\sigma_{c}^{-1}$.
— Cutting force should be sensitive to mandible geometry only if $R\geq G_{c}\sigma_{c}^{-1}$.

In testing these predictions, we hope to ultimately identify some of the key mechanical and morphological traits that modulate herbivore efficacy. What makes insects with biting–chewing mouthparts mechanically efficient, and how can plants best mechanically defend themselves?

## 2. Material and methods

### 2.1. Study animals

**Figure 1.**
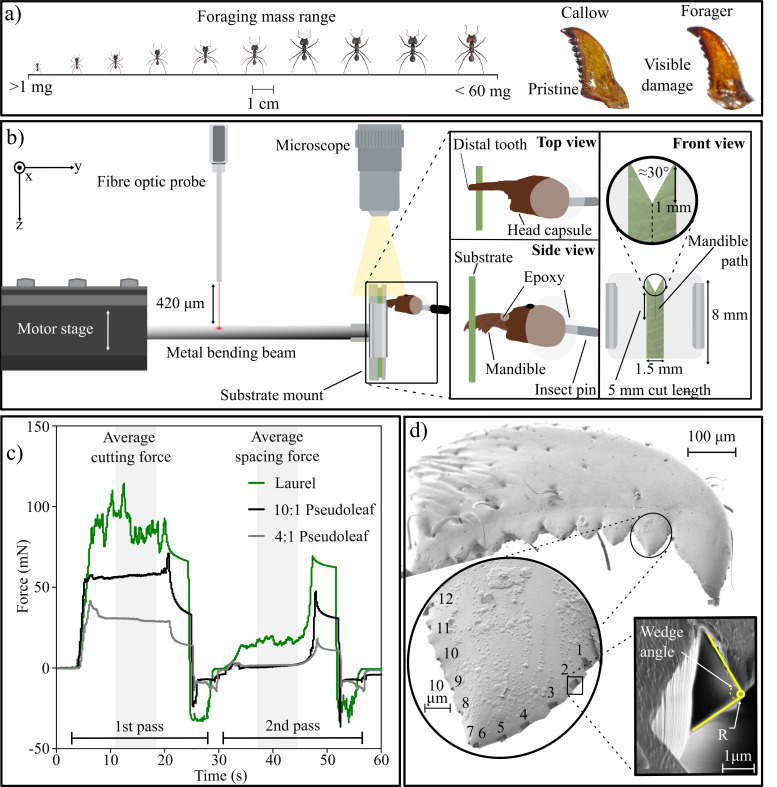
(a) The force required to cut biological and synthetic substrates was measured with mandibles extracted from *A. cephalotes* workers. To quantify the effects of mandibular wear and worker size on cutting force, mandibles were collected from both callows (with pristine mandibles) and foragers (with worn mandibles). (b) Cutting forces were measured using a custom-built set-up comprising a fibre-optic displacement sensor and a bending beam, both connected to a piezo motor stage. Test substrates were fixed in place using a custom-designed holder, mounted at the free end of the beam, and the mandible was positioned above the substrate such that its cutting edge was perpendicular to the plane of the substrate. The motor then moved the beam vertically upwards against the mandible, causing the substrate to be cut and the beam to deflect. (c) Illustrative cutting force traces, recorded continuously throughout both the first pass and the second pass. The total cutting force was measured in a first pass; a second pass through the cut was performed to extract spacing forces arising from side wall friction and elastic sheet bending; the difference between these two forces is the fracture force. Following cut initiation, cutting forces typically reach a steady state; the average forces across this area were used for further analysis. Force traces are from measurements for a callow mandible, extracted from a worker with body mass of 33.7 mg. (d) Mandible edge geometry was assessed by measuring cutting-edge radius and wedge angle from SEM images of cross-sections, cut with FIB milling such that they were approximately perpendicular to the cutting edge (*n* = 8, *n* = 6 and *n* = 1 for callow and forager mandibles and scalpel blades, respectively).

To test the two model predictions, we selected leaf-cutter ants as a model organism for three key reasons. First, they are the principal ecosystem engineer and the dominant insect pest species of the Neotropics, responsible for 15% of the total defoliation in the region and causing millions of pounds in annual crop damage—they are both ecologically and economically significant [43–45]. Second, they have a well-defined cutting method, using one mandible as an anchor while the other slices through the leaf lamina like a knife—a process that can be replicated experimentally [28,46–48]. And third, leaf-cutter ants are highly polymorphic, but show only minor mandible shape changes, providing an opportunity to vary mandible size and with it likely the cutting-edge radius R, without a covariation in shape [28,49]. *Atta cephalotes* (Linnaeus, 1758) leaf-cutter ants were sampled from a mature laboratory colony kept in a climate-controlled chamber (FitoClima 12.000 PH, Aralab, Rio de Mouro, Portugal) at 25°C, 60 ± 5% relative humidity and in a 12 h day/night cycle. The colony was provided with bramble, Japanese laurel, kibbled maize and honey water ad libitum. To measure the force required to cut biological and synthetic substrates with ant mandibles, mandibles were collected from workers spanning the typical size range of foraging parties, 1−60 mg; workers with body masses below 1 mg (minims) and above 60 mg (majors) were excluded, as they do not usually partake in foraging and have a distinctively different head and mandible morphology [29,49–51]. Two groups of workers were collected: workers with ‘pristine’ mandibles, allowing us to quantify the variation of mandible cutting forces with worker size independent of a possible covariation of mandible wear, and active foragers, to investigate the effect of mandible wear on cutting forces (figure 1a). Pristine mandibles (*n* = 27) were collected from callows—recently eclosed workers distinguished from mature workers by their softer and lighter cuticle (a detailed description of the collection process can be found in the electronic supplementary material and in [52,53]; see also [28,48]). Callows typically do not leave the colony nest and are physically incapable of leaf cutting in the first few days after eclosion [52]; their mandibles are therefore likely pristine, i.e. unaffected by wear induced by repeated cutting [28,48]. Forager mandibles (*n* = 20) were collected from mature workers, sampled from the foraging area such that their body masses were approximately evenly spaced in log_10_-space. The mandibles of these individuals are likely worn to varying extents, due to repeated leaf cutting ([28,48], see also [54–56] for similar reports in other species).

### 2.2. Mandible preparation

All individuals were weighed to the nearest 0.1 mg (Explorer Analytical EX124, max. 120 g × 0.1 mg, 130 OHAUS Corp., Parsippany, NJ, USA). Body masses ranged between 1.9 and 55.5 mg for callows and between 2.3 and 54.8 mg for foragers, representing the approximate size range that typically partakes in foraging activities [51,57,58]. Ants were then sacrificed by freezing at −4°C for at least 12 h, placed in a Petri dish, secured in place with Blutac (Bostik Ltd, Stafford, UK) and dissected under a light microscope (SAPO, Leica, Wetzlar, Germany). The head capsule was removed using micro scissors and split along the sagittal plane, using fresh scalpel blades (no. 11 blades, Swann Morton, Sheffield, UK). The head capsule of leaf-cutter ants is bilaterally symmetric [29], and ants do not appear to exhibit any obvious mandible laterality during cutting [47,59]. We hence assume that there are no systematic differences between each head capsule half [28,49]. The left side of the head capsule was retained for cutting force measurements; the right side was used for imaging and mandible cutting-edge radius measurements (see §2.6). To facilitate cutting force measurements, head halves were mounted on insect pins (size ‘2’ for ants < 10 mg, size ‘4’ for ants of 10−20 mg and size ‘6’ for ants > 20 mg; Shigakontyu, Tokyo, Japan). The connections between the pin and head, and the head capsule and mandible, respectively, were immobilized with two-component epoxy (Araldite Rapid, Huntsman 143 Corp., The Woodlands, TX, USA), taking great care that the epoxy did not cover the mandibular cutting edge, as confirmed via light microscopy. Nevertheless, some samples had to be discarded, reducing the sample size to *n* = 15 for both pristine and forager mandibles.

### 2.3. Model substrates

#### 2.3.1. Polydimethylsiloxane pseudoleaves

To provide well-defined test substrates, pseudoleaves were fabricated from polydimethylsiloxane (PDMS), which brings the advantage that all relevant structural and mechanical properties can be controlled and tuned through variation of the following four parameters: (i) the ratio of base and curing agent; (ii) sheet thickness; (iii) curing temperature; and (iv) curing time [60–64]. PDMS pseudoleaves were synthesized from Sylgard184™ (Dow Inc., Midland, MI, USA) [65], a two-component kit containing a vinyl-terminated base (A) and a curing agent (B) [60]. Pseudoleaves were fabricated with either a 10 : 1 or 4 : 1 mixing ratio. Films were made via a sandwiching method as described previously [28](see also electronic supplementary material). To further tune substrate mechanical properties—as required for testing the two leading hypotheses—curing conditions differed between the two mixing ratios. 10 :1 PDMS pseudoleaves were cured at 65°C for 4 h in a drying oven (Sanyo Electric Co., Ltd., Osaka, Japan) [66]; 4 : 1 PDMS pseudoleaves were instead cured at 100°C for 2 h and received a further 48 h post-cure at 165°C [60]. Four pseudoleaf samples, each with a size of approximately 95 cm^2^, were produced for each pseudoleaf type, each with two thicknesses (200 and 400 µm, respectively). We thus fabricated four pseudoleaf types: 200 µm 4 : 1 (thin, stiff and brittle), 200 µm 10 : 1 (thin, stiff and tough), 400 µm 4 : 1 (thick, compliant and brittle) and 400 µm 10 : 1 (thick, compliant and tough; see electronic supplementary material, figure S1). A total of 16 independently manufactured PDMS pseudoleaves were used. The thickness of each pseudoleaf was measured with a digital micrometer at six random locations (resolution 0.001 mm; Mitutoyo Corp, Kawasaki, Japan). The pseudoleaves had an average thickness of 210 ± 10 and 420 ± 15 µm, respectively, within 5% of the target thickness and with a variation of less than 5% about the mean thickness. Rectangular cutting test specimens (10 × 15 mm) were cut from each PDMS sheet using scalpel blades and kept in labelled and closed Petri dishes to avoid dust contamination until further use.

#### 2.3.2. Plant leaves and petals

Three plant tissues—Japanese laurel leaves (*Aucuba japonica*), bramble leaves (*Rubus fruticosus*) and rose petals (*Rosa sp*.)—were used to test whether insights from pseudoleaves apply in a biologically relevant context. The three plant tissues were selected because of a difference in thickness and perceived toughness: Japanese laurel leaf lamina is thick and sturdy, rose petals are thin and fragile and bramble leaves lie between the two. Leaves of bramble and Japanese laurel were collected from a private garden on Imperial College campus grounds (51.50078° N, −0.17242° W), between 8 February 2023 and 2 May 2023. Leaves were harvested at 10.00 ± 2 h on each experimental day, kept refrigerated in a sealed container and wrapped in damp tissue to minimize water loss [67]; all leaves were tested within 4 h of storage. Leaf mechanical properties can vary with leaf age [22,67–71]. To reduce such covariation, leaves were collected from the same bush and, where possible, from the same branches. We identified newly sprouting leaves, marked them and allowed them to mature for approximately two weeks before collection. Within each branch, leaf colour served as a qualitative proxy for maturity [72]; only leaves with dark green colouration were used in experimental trials [72]. Rose petals were bought from local suppliers, used on the day of purchase and stored as described above. A thickness map for each leaf lamina or petal was created by taking six thickness readings at random locations with a digital micrometer; the thickness of mid and minor veins was also recorded as present. The leaves had an average laminar thickness of 317 ± 38, 272 ± 14 and 188 ± 13 µm for Japanese laurel, bramble and rose petals, respectively. 10 × 15 mm rectangular test samples were cut from each biological substrate using scalpel blades, such that the sample’s long axis was perpendicular to the central leaf vein. Care was taken to select leaf areas free of minor or major veins to maximize substrate homogeneity [73]. Leaf-cutter ants typically avoid cutting major veins [74,75], so that measurements on more homogeneous sections of the leaf laminae are likely representative of the most frequent mechanical challenge [76,77].

#### 2.3.3. Mechanical characterization

In order to test the two hypotheses derived from the biomechanical model, three mechanical properties were determined for both pseudoleaves and real leaves where possible: (i) the Young’s modulus, E; (ii) the ultimate tensile strength (UTS), σ; and (iii) the tearing energy, $G_{c}$. The mechanical characterization of homogeneous materials such as PDMS is comparatively straightforward. Biological materials, however, pose a number of challenges. First, on smaller leaves and petals, it is often impossible to cut samples with dimensions that comply with International Organization for Standardization/American Society for Testing and Materials (ISO/ASTM) standards; miniaturized or non-standard geometries must be used instead, which compromises comparability. Second, biological materials such as leaves are often heterogeneous, so that standard assumptions including those of linear elastic fracture mechanics may not hold [78,79]. These difficulties prevented mechanical characterization for rose petals and bramble leaves, which were either too small or too heterogeneous to yield consistent test results, respectively. For each material parameter, two test samples were taken from each of four pseudoleaf sheets (*n* = 8 per pseudoleaf condition, *n* = 32 total), unless stated otherwise. Twenty Japanese laurel leaves were used across this study; three samples per leaf were taken for each test. All tests were conducted with a uniaxial tensile testing machine, equipped with Mec277 double-action vice grips with diamond jaws (Multitest5-xt, Mecmesin Ltd., Slinfold, UK; 25 N load cell, ± 0.25N resolution). All samples were stretched until failure while measuring load and displacement. From these measurements, Young’s modulus, UTS and tearing energy were all obtained using standard techniques [39,80–82] (see electronic supplementary material for details). All pseudoleaf material properties were independent of pseudoleaf thickness (two-way ANOVA; Young’s modulus: *F*_1,28_ = 0.164, *p* = 0.689; UTS: *F*_1,20_ = 0.003, *p* = 0.960; tearing energy: *F*_1,28_ = 0.787, *p* = 0.954) but varied significantly with mixing ratio (Young’s modulus: *F*_1,28_ = 609.1, *p* < 0.001; UTS: *F*_1,20_ = 5.40, *p* = 0.048; tearing energy: *F*_1,28_ = 222.6, *p* < 0.001). Results were thus pooled for each thickness. Material properties for each pseudoleaf type and Japanese laurel are presented in table 1.

**Table 1.** Cutting forces are expected to vary with the mechanical properties of the cut material, which were thus characterized. Young’s modulus and ultimate tensile strength (UTS) were determined via tensile testing of dog-bone specimens, and tearing energy was measured via pure shear tests. The Young’s modulus—the ratio between stress and strain in the linearly elastic region—is a proxy for the material’s resistance to elastic deformation; the UTS represents the maximum stress a material can withstand; and the tearing energy denotes the energy per unit area of crack surface. The ratio between the tearing energy and the UTS yields a characteristic length scale [28,39,41]; cutting tool geometry is irrelevant provided that a characteristic tool length scale, for example, the cutting-edge radius, is small compared to this length. Material properties were independent of sheet thickness for PDMS pseudoleaves and were thus pooled. All values represent mean and s.d., and references in square brackets provide independent measurements of these properties in agreement with our data.

substrate	Young’s modulus *E* (MPa)	tearing energy *G*_*c*_ (J m^−2^)	UTS $\sigma_{c}$ (MPa)	material length scale (µm), *G*_*c*_/$\sigma_{c}$
4 : 1 PDMS pseudoleaf	4.1 ± 0.3 [39]	98 ± 9 [39]	6.0 ± 2 [83]	16.3 ± 5.6
10 : 1 PDMS pseudoleaf	1.6 ± 0.3 [60]	197 ± 25 [60]	5.5 ± 2 [83]	35.8 ± 13.8
Japanese laurel	31 ± 4	746 ± 210	1.63 ± 0.3	449 ± 153.9

### 2.4. Cutting force set-up

The forces required to cut both pseudoleaves and real leaves with ant mandibles were measured with a custom-built set-up, based on a fibre-optic displacement sensor (μDMS-RC32 controlled via DMS Control v3.015; Philtec Inc., Annapolis, MD, USA; see figure 1b and [28,84]). The sensor was fixed in place with a custom mount attached to two micromanipulators, providing control over its orientation, such that it was perpendicular to a stainless-steel bending beam placed below. The beam (114.7 mm (L) × 10.4 mm (W) × 0.35 mm (T)) was placed so that the sensor remained within its linear range of 2.5 mm throughout the measurements—420 µm above the beam in its undeformed state (figure 1b). The beam was held in place by a metal plate, leaving a free length of 28.7 mm. The plate holding the beam was attached to a movable motor stage (M-404.6PD, controlled via PIMikroMove v 2.33.3.0; Physik Instrumente GmbH & Co. KG, Karlsruhe, Germany; maximum velocity 25 mm s^−1^). At the free end of the beam, a custom-designed three-dimensional (3D) printed mount was fitted to constrain the substrates during measurements. This mount consisted of two parts, clamped together using metal clips (Supaclip 40, Rapesco Office Products PLC, Sevenoaks, UK). A more detailed description of this set-up and sensor calibration may be found in [28] and the electronic supplementary material.

### 2.5. Cutting force measurements

Before each experimental trial, substrates were ‘pre-cut’ using a scalpel blade and 3D printed template, resulting in a wedge with an opening angle of about 30° and a depth of about 1 mm (figure 1b). This pre-cut decreases the propensity for substrate bending and buckling and thus aids cut initiation, but it leaves the steady-state cutting force unaffected [39,46,85]. Substrates were then clamped with the pre-cut centred in a 1.5 mm (W) × 8 mm (H) free region for mandibles and a wider 5 mm (W) × 8 mm (H) free region for scalpel blades to accommodate their larger size. Preliminary trials were conducted to assess the appropriate size of the free region (electronic supplementary material, figure S3). Prepared mandibles were first clamped with an insect pin holder, which was then attached to a 3D micromanipulator (MM 33, Märzhäuser Wetzlar, Germany), and positioned such that the apical tooth of the mandible was roughly perpendicular to the substrate and just about extended over the sheet edge; mandible orientation was aided and verified with a custom-built top-down microscope. Previous work confirmed that the cutting force does not significantly vary between repeated mounts of the same sample, i.e. that this mounting procedure is repeatable [28]. Pseudoleaves were thus measured only once per sample [28]; biological substrates, however, also bring material inhomogeneity and were thus measured three times with each mandible. All mandible cutting force measurements were performed in a randomized order to minimize systematic effects of mandible wear on substrate-specific cutting forces. Once the substrate and mandible were aligned, the substrate was moved upwards against the mandible to initiate cutting. The stage movement proceeded at a speed of 0.3 mm s^−1^ for 5 mm during each trial, sufficient to initiate the cut and to achieve steady-state cut propagation, as indicated by an approximately constant force reading (figure 1c). The stage movement speed was chosen to be comparable to the cutting speed of larger foragers [28,50,86]. Cutting forces do vary with cutting speed, but this effect is typically small and thus not further evaluated here [87]: an increase in cutting speed by a factor of three increases cutting forces by only about 20% [28]. Each measurement consisted of two passes. The first pass was used to determine the total cutting force ($F_{\text{c}}$); the second pass through the now cut sheet was used to determine the contribution from spacing forces ($F_{\text{s}}$) due to elastic sheet deformation and sidewall friction (figure 1c) [28,88–90]. Measurements were considered invalid and thus repeated if one of the following occurred: (i) contact between the head capsule/epoxy and the cutting substrates; (ii) failure of the epoxy fixation at the joint; (iii) damage/destruction of the mandible; (iv) the steady-state region was too short to extract a meaningful cutting force (less than 2 mm cutting length). In total, we conducted *n* = 390 mandible cutting force measurements: 2 × 15 mandibles for three natural substrates with three repeats, plus for four synthetic substrates with no repeats. For each substrate, control measurements were also conducted using pristine scalpel blades. A fresh scalpel was used for each substrate (seven scalpels total); repeats were conducted as above, giving a total of *n* = 13 measurements (see electronic supplementary material for details).

### 2.6. Mandible cutting-edge geometry

To characterize the geometry of the mandible cutting edge, its edge radius and wedge angle were measured using focused ion beam milling and scanning electron microscopy (FIB and SEM) [91–94]. A subset of eight callow and six forager mandibles were selected from the dissected right head capsule halves (see §2.2) such that their body masses were distributed evenly across the sample mass range in log_10_-space; the smaller sample size reflects the time-intensity of FIB milling. Mandibles were fixed onto atomic force microscopy (AFM) sample holders under a stereomicroscope, using Everbuild high viscosity superglue (Sika, Baar, Switzerland). They were positioned at the edge of the AFM stub to prevent collisions with the FIB column and oriented such that the dorsal side of the mandible faced upwards, and the mandible teeth faced the edge of the stub. Samples were then sputter-coated with 40 nm of chromium (Q150Tplus, Quorum, Sussex, UK). To obtain sections approximately perpendicular to the cutting edge, FIB milling was conducted using a Zeiss Auriga FIB–SEM at 30 kV acceleration voltage and 240 pA current. The voltage was fixed to reduce damage to the samples, and the current was optimized via trial and error to obtain clean sections. A total of 12 trapezoidal cuts were milled on the second tooth of each mandible (counted from distal); six cuts were made on each side (figure 1d), distributed from the base to the tip of the tooth (figure 1d). Of these six cuts, one was taken in the bottom 20% of the tooth height; the rest were distributed along the top 80%; the cuts were thus symmetric about the tooth centre, but not evenly distributed in height (figure 1d). The cutting-edge radius and wedge angle of scalpel blades were also characterized. Scalpel blades, manufactured with uniform geometry, were represented by a single blade, with five evenly distributed cuts made along its edge. Preparation, mounting and imaging conditions were as described above. SEM imaging was conducted on the same machine at an acceleration voltage of 3 kV. Images were taken such that an equivalent planar view on the exposed edge cross-section was ensured across all milled areas (figure 1d). Subsequent analysis was completed in ImageJ (1.54f) [95]. Wedge angle was defined by placing two lines along the straight part of the cutting edge and then measured utilizing the inbuilt angle tool. Cutting-edge radius was defined as the radius of the smallest circle tangential to the two lines that still fits within the cross-section (figure 1d; see [96] for a similar methodology).

### 2.7. Data curation and analysis

Raw data obtained from the fibre-optic sensor (.txt file) were converted to obtain force–time data, from which average total cutting force and spacing force were extracted for a 2 mm steady-state region after the initial peak (figure 1c).

Fracture force (*F*_c_) was determined by removing the contribution of the spacing force (*F*_s_) from the total cutting force (*F*_c_), $F_{\text{f}}=F_{\text{c}}-F_{\text{s}}$. Cutting force measurements conducted on biological substrates were corrected for lamina thickness (*t*_1_), $F_{c}=\frac{F_{c,c}\bar{t_{1}}}{t_{1}}$, where ${\bar{t}}_{1}$ is the average laminar thickness, and *F*_c,c_ is the uncorrected cutting force [28]. All these steps were conducted in Python (v. 3.9.7).

The relationship and interaction between worker body mass, pseudoleaf type, substrate thickness and mandible group were analysed via a repeated measures analysis of covariance (RM ANCOVA). A nested RM ANCOVA was used to assess the relationship between worker body mass, mandible group and leaf type. Type II sum of squares permitted us to accurately assess main effects in the presence of possible interactions [97]. Log-transformed measured force (*F*_c_, *F*_f_ or *F*_s_) was the continuous response variable, and body mass (continuous, log-transformed), group (categorical), mixing ratio (categorical) and thickness (categorical) or body mass (continuous, log-transformed), plant type (categorical) and group (categorical) were the predictor variables for pseudoleaves and plant tissues, respectively. Sample (mandible ID) was included as a random effect to account for repeated measures across substrates in pseudoleaves; for measurements in plant tissues, sample (mandible ID) was treated as a nested random factor in a nested RM ANCOVA model to account for within-substrate repeats. We verified that RM ANCOVA assumptions, including normality of residuals (Shapiro–Wilk test), sphericity and homoscedasticity (Mauchly’s test, visual inspection), were met.

Ordinary least squares regressions on log_10_-transformed data were performed to characterize scaling relationships between response variables and body mass. The assumptions of the linear model were verified as outlined above. Values stated in text and tables are mean ± s.d. unless stated otherwise; boxplots presented show the sample median; whiskers extend to 1.5 times the interquartile range from the first and third quartile; and outliers are plotted beyond this range. All statistical analysis and plotting were conducted in R (v. 4.1.1).

## 3. Results

### 3.1. Testing model predictions using pseudoleaves

#### 3.1.1. Cutting force is proportional to thickness, independent of material length scale or mandible size

**Figure 2.**
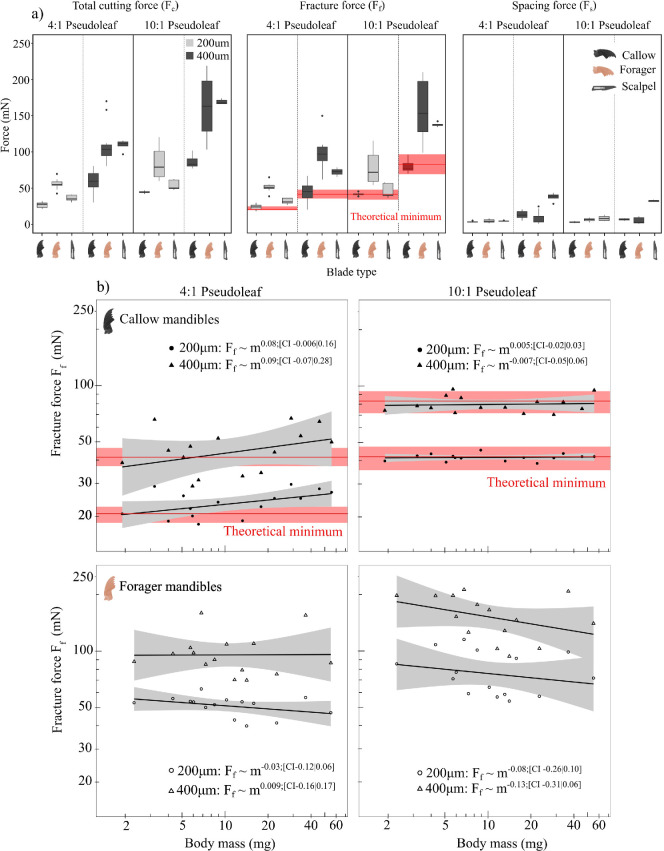
To investigate the relationship between cutting force and worker body mass, mandible wear, pseudoleaf thickness and pseudoleaf type, we measured total cutting, fracture and spacing forces across different pseudoleaves for two mandible groups: callow mandibles that are pristine, and forager mandibles with varying degrees of mandible wear (*n* = 15 for both). Measurements were conducted on four pseudoleaf types that varied in thickness and toughness: 200 µm 4 : 1 PDMS, 400 µm 4 : 1 PDMS, 200 µm 10 : 1 PDMS and 400 µm 10 : 1 PDMS. Red lines show the theoretical minimum force to fracture each substrate, predicted from the sheet thickness t and toughness $G_{c}$ (equation (1.1)). Error bounds, calculated via error propagation, are shaded. (a) Total cutting, $F_{\text{c}}$, and fracture forces, $F_{\text{f}}$, varied significantly with mandible group, thickness and pseudoleaf type. On average, $F_{\text{c}}$ and $F_{\text{f}}$ of callow mandibles were about half those of forager mandibles, and a twofold increase in thickness corresponded to a proportional increase in force for both pseudoleaf types and mandible groups. Cutting force also nearly doubled between 4 : 1 and 10 : 1 pseudoleaf types. Scalpel blade forces were close to those of callow mandibles for thin films, but close to those of foragers for thick films. All boxplots display the median (centre line), the first and third quartiles (hinges), extended by 1.5 times the interquartile range (whiskers), and outliers (points). (b) All forces were independent of worker body mass and thus mandible size. However, there was a weak trend such that forces increased with body mass for callow mandibles, but decreased with body mass for forager mandibles.

Total cutting force, $F_{\text{c}}$, increased significantly with pseudoleaf thickness (RM ANCOVA: *F*_1,26_ = 341.32, *p* < 0.001), an effect independent of worker body mass, pseudoleaf type (4 : 1 versus 10 : 1) and mandible group (callow versus forager; see electronic supplementary material, table S2). As predicted, $F_{\text{c}}$ was directly proportional to pseudoleaf thickness for both mandible groups, i.e. a twofold increase in thickness approximately doubled the cutting force (electronic supplementary material, table S1, and figure 2a). $F_{\text{c}}$ however differed significantly between the two groups (RM ANCOVA: *F*_1,26_ = 386.6, *p* < 0.001): forager mandibles required about twice the cutting force than pristine mandibles for all pseudoleaf types, independent of worker body mass (figure 2a). In other words, forager mandibles cut a 200 µm pseudoleaf with about the same force as callow mandibles cut a 400 µm pseudoleaf. Spacing forces, $F_{\text{s}}$, accounted for up to 12 and 22% of the total cutting forces—or 4 ± 2 and 16 ± 12 mN—for thin and thick pseudoleaves, respectively (RM ANCOVA, thickness: *F*_1,26_ = 11.9, *p* = 0.001; figure 2a). Accordingly, fracture forces, $F_{\text{f}}$, mirrored the results for $F_{\text{c}}$: they increased significantly with pseudoleaf thickness (RM ANCOVA: *F*_1,26_ = 306.2, *p* < 0.001), but this effect was independent of worker body mass, pseudoleaf type and mandible group (see electronic supplementary material, tables S1–S4). Scalpel blade total cutting forces were comparable to those of callow mandibles for thin films (200 µm), but close to forager mandibles for thick films (400 µm), a trend that held for both pseudoleaf types (figure 2a and electronic supplementary material, table S1). $F_{\text{s}}$ also differed between film thicknesses; they were 6± 4 and 35 ± 6 mN for 200 and 400 µm pseudoleaves, respectively. These forces were comparable to both mandible groups for thin films, but were significantly larger for thick films, contributing 13 and 27% to the total cutting force, respectively. To confirm statistically that the variation in cutting force indeed reflects solely an effect of thickness, we divided the cutting and fracture force of each sample by the average thickness of the test specimen and then compared the thickness-corrected forces. An RM ANCOVA revealed no significant difference in force after thickness correction, indicating that thickness was the primary driver of differences in force across pseudoleaves made with the same mixing ratio. (RM ANCOVA: $F_{\text{c}}$, *F*_1,26_ = 0.455, *p* = 0.51, $F_{\text{f}}$: *F*_1,26_ = 0.117, *p* = 0.74). The thickness-corrected force also remained dependent on mandible group and still differed significantly across mandible groups and pseudoleaf types (RM ANCOVA: mandible group $F_{\text{c}}$, *F*_1,26_ = 138.26, *p* < 0.001, mandible group *F*_1,26_ = 133.1, *p* < 0.001, pseudoleaf type $F_{\text{c}}$, *F*_1,26_ = 192.82, *p* < 0.001, pseudoleaf type $F_{\text{f}}$, *F*_1,26_ = 240.67, *p* < 0.001; electronic supplementary material, tables S5 and S6).

#### 3.1.2. Pristine mandibles can be considered ideally sharp: their fracture forces approach a physical minimum

In order to place the magnitude of the cutting force into perspective, an approximate theoretical prediction may be derived via a simple virtual work argument [36,39–41] (see equation (1.1)). This simple model suggests that a direct estimate for a lower bound for the cutting force is given by the product between sheet thickness, t, and toughness, $G_{c}$ alone. Because these are experimental quantities, they are associated with an error, and the bounds on the estimate were thus calculated as propagated uncertainty following [98]. Fracture forces of callow mandibles consistently fell within the theoretical minimum bands for all pseudoleaf types, indicating that callow mandibles are ideally sharp [40]; there was no statistically significant difference between the calculated physical minimum force and the measured forces with callow mandibles (one way repeat measures ANOVA: *F*_1,14_ = 0.25, *p* = 0.63; figure 2 and electronic supplementary material, table S7).

#### 3.1.3. Material length scale affects the allometry of cutting force

Young’s modulus, UTS and tearing energy differed significantly across the two pseudoleaf types—they thus have a different material length scale (see §2.3 and table 1). Total cutting forces, $F_{\text{c}}$, depended significantly on pseudoleaf type (RM ANCOVA: *F*_1,26_ = 157.03, *p* < 0.001): as predicted, a factor of two increase in toughness resulted in a proportional increase in force. A mandible from the same group thus cut a 400 µm 4 : 1 film with roughly the same force as a 200 µm 10 : 1 film (figure 2a). $F_{\text{c}}$ was, however, independent of worker body mass, regardless of mandible group, pseudoleaf type and thickness (figure 2a and electronic supplementary material, table S2). Despite this absence of a main effect, there was a significant interaction between pseudoleaf type and worker body mass (electronic supplementary material, table S2), suggesting that the mass scaling exponent, however small, differs significantly between pseudoleaf types. This result, however, must be interpreted with caution: fracture forces also depended significantly on pseudoleaf type (RM ANCOVA: pseudoleaf type *F*_1,26_ = 181.5, *p* < 0.001), but showed no significant interaction (electronic supplementary material, table S3). Although the main effect of worker mass was statistically insignificant, a clear trend was visually discernible. For callows, $F_{\text{c}}$ showed a small but statistically insignificant trend to increase with body mass for 4 : 1 pseudoleaves, but was almost perfectly size invariant for 10 : 1 pseudoleaves, which have a material length scale almost twice as large (figure 2b). For forager mandibles, in turn, $F_{\text{c}}$ were approximately constant with respect to worker body mass on 400 µm 4 : 1 pseudoleaves, but showed an insignificant trend to decrease with size on 200 µm 4 : 1 and 10 : 1 pseudoleaves regardless of their thickness (figure 2b). Spacing forces were independent of worker body mass across all tested conditions (electronic supplementary material, table S4).

### 3.2. Biological substrates

#### 3.2.1. Cutting force varies with perceived plant toughness, independent of mandible size

**Figure 3.**
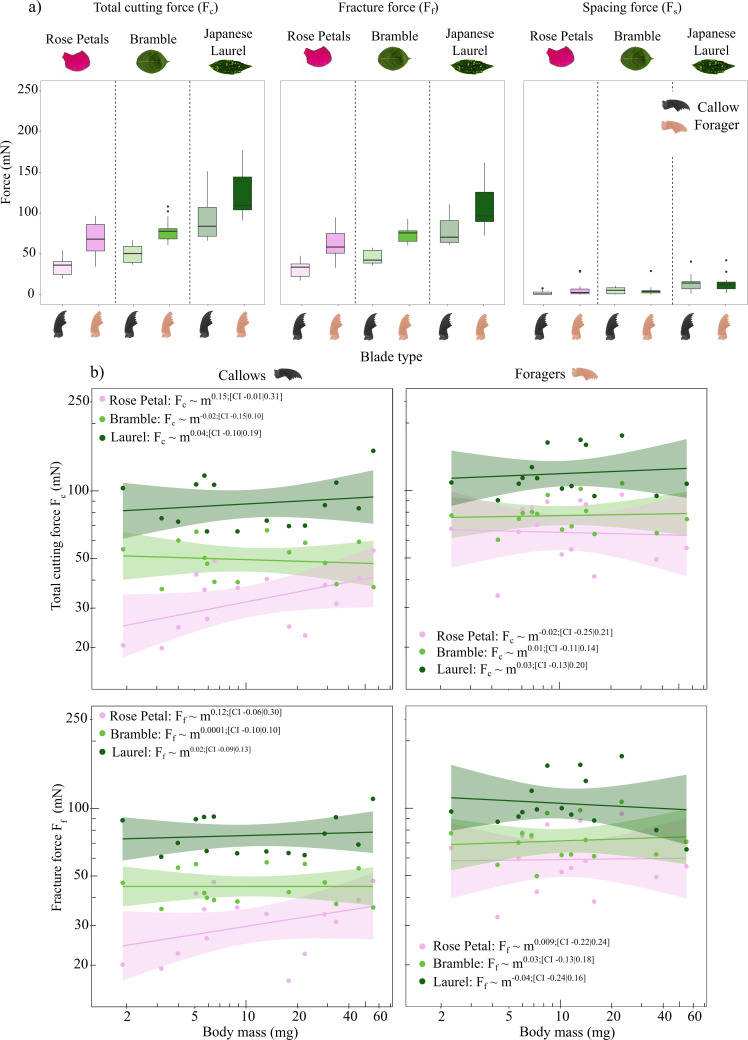
Total cutting ($F_{\text{c}}$), fracture ($F_{\text{f}}$) and spacing ($F_{\text{s}}$) forces were measured for three natural substrates: rose petals, bramble and Japanese laurel leaf lamina. For each substrate, cutting forces were measured for two mandible groups: pristine callow mandibles and forager mandibles, which showed variable degrees of wear (*n* = 15 per group, three repeats per leaf, *n* = 270). Forces were re-scaled to average leaf thickness. (a) $F_{\text{c}}$ depended significantly on the mandible group and plant type. For rose petals, callow mandibles cut with almost half the force measured for forager mandibles. For bramble and Japanese laurel, the difference was smaller, but consistent in direction. $F_{\text{f}}$ largely mirrored these trends; $F_{\text{s}}$ varied across plant materials, but not with mandible group, and their overall contribution to $F_{\text{c}}$ was a mere 9–14%. (b) All forces were independent of worker body mass and thus of mandible size. However, forces tended to increase with body mass for callow mandibles and to decrease with body mass for forager mandibles, a trend most pronounced in rose petals.

Cutting force, $F_{\text{c}}$, varied significantly with plant type (RM ANCOVA ($F_{\text{c}}$): *F*_1,26_ = 404.7, *p* < 0.01; see electronic supplementary material, table S9). In line with the perceived toughness, they were highest for Japanese laurel and lowest for rose petals (91 ± 24 and at 34 ± 11 mN, respectively; data for callow mandibles, see figure 3a and electronic supplementary material, table S8). Fracture forces, $F_{\text{f}}$, also depended significantly on plant type (RM ANCOVA plant type: *F*_1,26_ = 125.5, *p* < 0.001; electronic supplementary material, table S10), essentially mirroring the results for $F_{\text{c}}$. Within callow mandibles, $F_{\text{f}}$ ranged from a minimum of 31 ± 11 in rose petals, over an intermediate value of 46 ± 11 mN in bramble, to a maximum of 78 ± 24 mN in Japanese laurel—a variation of a factor of about 2.5 (see figure 3a, electronic supplementary material, tables S8–S10). Mandible spacing forces ($F_{\text{s}}$) varied across plant types (RM ANCOVA $F_{\text{s}}$ plant: *F*_1,26_ = 22.03, *p* < 0.001; see electronic supplementary material, table S11). As for pseudoleaf cutting experiments, this effect was small: spacing forces accounted for a mere 9–14% across all plant materials and mandible groups (figure 3a). None of the measured forces $F_{\text{c}}$, $F_{\text{f}}$, $F_{\text{s}}$ varied significantly with worker body mass and therefore mandible size (RM ANCOVA body mass: $F_{\text{c}}$, *F*_1,26_ = 0.77, *p* = 0.39, $F_{\text{f}}$, *F*_1,26_ = 0.34, *p* = 0.57, $F_{\text{s}}$, *F*_1,26_ = 0.1, *p* = 0.80; figure 3b). However, there were some notable allometric trends. In rose petals, there was a weak but insignificant trend for forces to increase with size for callow mandibles and to decrease with size for foragers, as observed earlier for pseudoleaves (callows: $F_{\text{c}}\propto m^{0.15}$, $F_{\text{f}}\propto m^{0.12}$; foragers: $F_{\text{c}}\propto m^{-0.02}$, $F_{\text{f}}\propto m^{0.0009}$). Allometric trends in bramble and Japanese laurel did follow the same pattern, but were more subtle (figure 3b). Despite an insignificant main effect of worker body mass on spacing forces, $F_{\text{s}}$, there was a significant interaction between worker body mass and plant type (electronic supplementary material, table S11); the scaling relationship of the spacing forces thus differed between plant materials.

#### 3.2.2. Mandible wear significantly increases cutting forces

Total cutting forces, $F_{\text{c}}$, varied significantly across mandible groups (RM ANCOVA ($F_{\text{c}}$): *F*_1,26_ = 43.51, *p* < 0.001; electronic supplementary material, table S9). For rose petals, the difference amounted to approximately a factor of two (68 ± 20 versus 34 ± 11 mN for forager and callow mandibles, respectively), resembling the results for pseudoleaves. However, the difference was less pronounced for bramble and Japanese laurel leaf laminae, where it amounted to factors 1.6 and 1.3, respectively (figure 3a and electronic supplementary material, table S9). Mandible spacing forces ($F_{\text{s}}$) did not differ significantly with mandible group (RM ANCOVA ($F_{\text{s}}$): *F*_1,26_ = 0.89, *p* = 0.36; electronic supplementary material, table S11); therefore, fracture forces, $F_{\text{f}}$, also depended significantly on mandible group (RM ANCOVA ($F_{\text{f}}$): *F*_1,26_= 40.34, *p* < 0.01; electronic supplementary material, table S10) .

### 3.3. Pristine mandible cutting-edge radii are size invariant, but increase significantly due to wear

**Figure 4.**
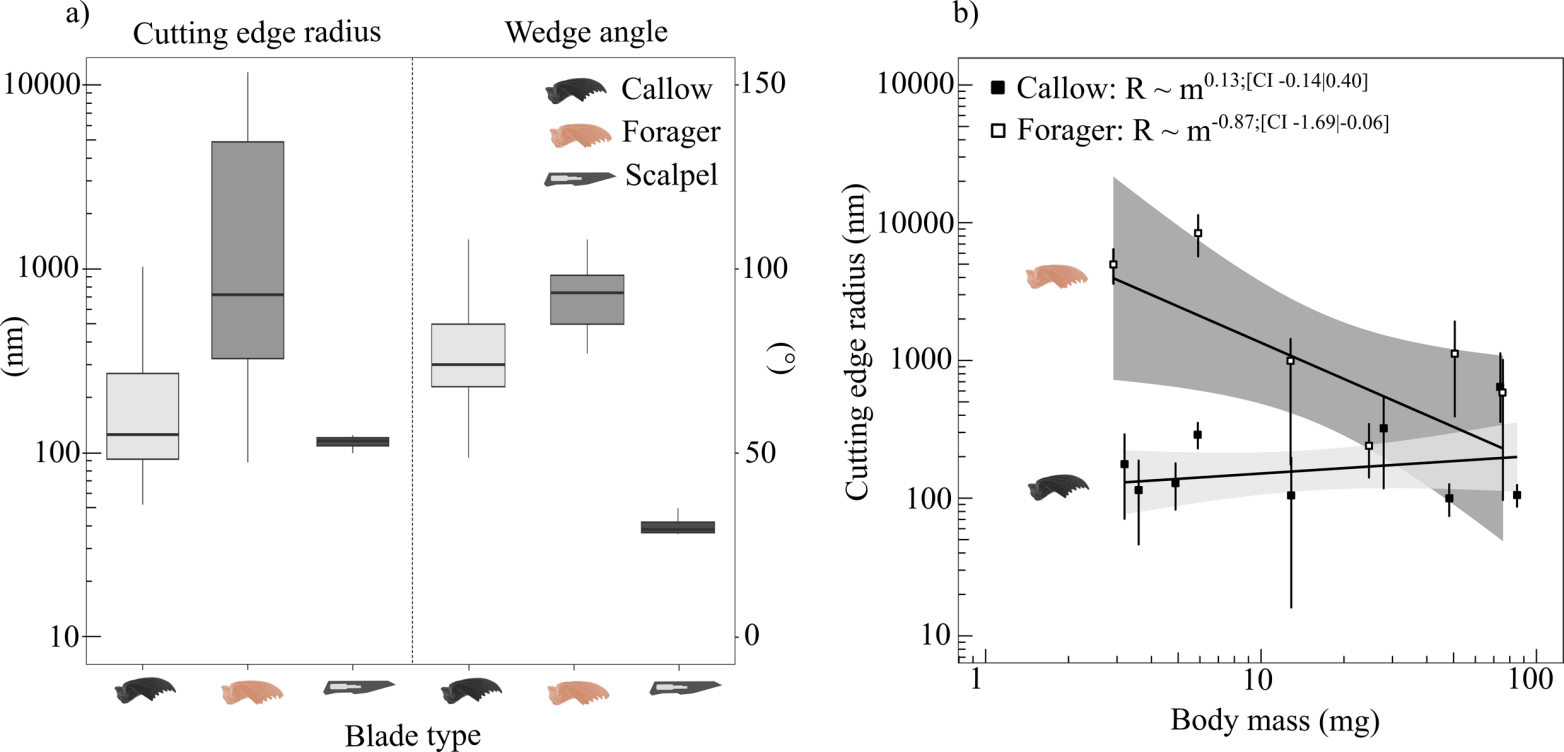
In order to assess the contributions of mandible geometry to cutting forces, we measured the cutting-edge radius R and wedge angle of pristine and forager mandibles; scalpel blades served as reference (*n* = 8, *n* = 6 and *n* = 1, respectively). (a) Cutting-edge radius varied significantly with mandible wear state—in the most extreme case, it differed by a factor of more than 200 (note the logarithmic *y*-axis on the left). The average cutting-edge radii were $R_{\text{c}}$= 162 ± 120 nm, $R_{\text{f}}$= 2530 ± 3300 nm and $R_{\text{s}}$= 115 ± 15 nm for pristine mandibles, forager mandibles and scalpel blades, respectively. Pristine mandibles were thus about as sharp as scalpel blades. The wedge angle varied minimally but significantly between callow and forager mandibles and was substantially higher than for scalpel blades. Boxplots display the median (centre line), the first and third quartiles (hinges), extended by 1.5 times the interquartile range (whiskers). (b) Callow cutting-edge radii showed an insignificant trend to increase with worker size ($R_{\text{c}}\propto m^{0.13}$). In contrast, forager mandible cutting-edge radii decreased significantly with mass ($R_{\text{f}}\propto m^{-0.87}$), suggesting that larger workers have less worn mandibles.

An RM ANCOVA revealed a significant effect of mandible size (mass) on combined cutting-edge radius for both callow and forager mandibles (RM ANCOVA: mass *F*_1,52_ = 5.42, *p* = 0.02). However, *post hoc* univariate ANOVAs revealed that callow mandible radii did not vary significantly with mass (ANOVA: *F*_1,52_ = 4.01, *p* = 0.09), so that this effect reflects solely a decrease of the edge radius with mandible size in forager mandibles (ANOVA: *F*_1,52_ = 15.01, *p* < 0.01). Across all mandible groups, tooth location had no significant effect on cutting-edge radius (ANCOVA: location *F*_1,52_ = 2.32, *p* = 0.08). Values were hence pooled to determine the average cutting-edge radius for pristine mandibles as $R_{c}$= 162 ± 120 nm (figure 4a). Forager mandible cutting-edge radius $R_{f}$ decreased significantly with worker body mass ($R_{\text{f}}\propto m^{-0.87}$), dropping by almost one order of magnitude: smaller workers appear to have more worn mandibles (figure 4b). The pooled average cutting-edge radius for forager mandibles was $R_{f}$= 2530 ± 3300 nm. From the most worn forager mandible to the smallest pristine cutting-edge radius, there was a 200-fold decrease in cutting-edge radius (electronic supplementary material, table S12). The cutting-edge radius of scalpel blades was $R_{s}$= 115 ± 15 nm, comparable to that of callow mandibles.

The wedge angle of the mandible cutting-edge did not vary significantly with worker body mass and tooth location (ANCOVA mass: $F_{1,46}=2.55,p=0.12$; ANCOVA location: $F_{1,46}=0.74,p=0.60$). It did, however, depend significantly on mandible group (ANCOVA mandible group: $F_{1,46}=13.4,p<0.01$). On average, the cutting-edge wedge angle was $77\pm 15^{\circ }$ and $93\pm 9^{\circ }$ for callow and forager mandibles, respectively (figure 4a)—a factor of two to three lower than for scalpel blades ($30\pm 3^{\circ }$; electronic supplementary material, table S13).

## 4. Discussion

Insects and plants have co-existed for over 350 million years [1]. Perhaps the most consequential of their interactions is that many insects feed on plants [3]. So ubiquitous is this interaction that virtually every plant species is consumed by at least one insect species [99], leading to an ongoing and widespread evolutionary arms race in which specialized feeding ‘tools’ evolved in insects, and specialized defence strategies evolved in plants. Much attention has been paid to chemical deterrents to herbivory [100–103], but only a much smaller number of studies discussed mechanical defences ( see [20,23,68,104–107]); the fundamental mechanical and morphological pressures on the insect feeding apparatus remain less understood still [13,16–18,29,40,48,52,108–111]. To learn more about some of the mechanical components of this ancient contest, we set out to test a simple physical model that relates the leaf-cutting force to the structural and mechanical properties of the cut material and the geometry of the cutting tool. Because leaves are complex and inhomogeneous composite materials, we initially tested the model with homogeneous pseudoleaves made from elastomers, giving us control over the relevant structural and mechanical properties. Because mandibles come in many complex shapes (e.g. [33]), introducing variation that is difficult to account for, we used mandibles from leaf-cutter ant workers as a model for blades that differ in size, but only a little in shape [49]. In the following discussion, we inspect the evidence in support of and against the simple model, evaluate the extent to which it can be applied to more complex plant tissues and then leverage these results to pinpoint strategies to make plant cutting as hard or as easy as possible, respectively.

### 4.1. The biomechanics of leaf cutting

Breaking up plant tissue involves the creation of new surface area; it may thus be conceptualized as a fracture process. Combining a simple virtual work argument with dimensional considerations yields a first-order prediction of the steady-state force required to blade-cut thin leaves (equation (1.3); see also [40–42,112]) and identifies two relevant regimes, separated by the ratio of two characteristic length scales: the radius R of the cutting-edge and the material length scale $G_{c}\sigma_{c}^{-1}$. The relative importance of these length scales may be determined by inspecting the dimensionless group $\Omega =C\sigma_{c}RG_{c}^{-1}$ [40,41], defined by this ratio.

For Ω<<1, $F_{f}\approx G_{c}t$—the steady-state fracture force is unaffected by blade geometry and determined solely by the properties of the cut material. Empirically, this scenario appears to apply to pristine mandibles (see also [40]), for which fracture forces were indistinguishable from this prediction, independent of whether pseudoleaf thickness, toughness or both were varied (figure 2). The fracture force corresponded to about 90% of the total cutting forces in all trials, indicating further that contributions from other energy sinks, subsumed in the spacing forces, are small enough to be neglected; these results justify the omission of other energy terms in the cutting energy balance *a posteriori*. Thus, and all else being equal, the cutting forces for pristine mandibles are dominantly defined by pseudoleaf thickness and toughness.

If Ω is comparable or much larger than unity, blade geometry comes into play [36,39–42]. Three experimental treatments were implemented to manipulate the magnitude of Ω and to thus probe the validity of the model: a variation in mixing ratio and curing conditions of the pseudoleaves, which altered the characteristic material length scale by about a factor two (table 1); a pristine mandible and forager mandible group, distinguished by a different likelihood of mandibular wear [40,48]; and a variation in mandible size, realized by using mandibles from foragers that differed by more than one order of magnitude in body mass.

Empirically, it would appear that Ω is comparable to unity for forager mandibles, for their fracture forces were consistently and significantly higher than the lower bound (figure 2). Three further observations are qualitatively consistent with this physical interpretation: the disparity between pristine, forager and scalpel blade fracture forces was independent of pseudoleaf thickness (figure 2); the disparity between pristine and forager mandibles was less pronounced for 10 : 1 than for 4 : 1 pseudoleaves, presumably because the larger material length scale of 10 : 1 pseudoleaves masked the effects of geometric differences (figure 2); and the difference between forager and callow fracture forces was smallest in Japanese laurel leaves, which had by far the largest material length scale. To render this qualitative discussion quantitative, we note that the fracture forces of pristine mandibles are within the uncertainty of the lower bound estimate $F_{f}=G_{c}t$ if Ω<0.05; the results for forager mandibles, in turn, demand Ω≈1. A closer assessment of the quantities R, $\sigma_{c}$ and C is in order.

R represents a characteristic length scale of the cutting tool. For a simple geometry such as a wire, the choice is obvious, for there is only one length scale [41,42,113]. But for a more complex geometry, such as an insect mandible, the case is much less clear cut. Using FIB milling, we measured the radius of the mandible cutting edge—not the only, but perhaps the most obvious choice for R [39]. In contradiction with the parsimonious hypothesis of geometric similarity, $R\propto m^{1/3}$, R took a size-invariant mean value of 162 nm for pristine mandibles. $\sigma_{c}$ is a quantity of dimension force per area, and for PDMS pseudoleaves, $\sigma_{c}\approx 6$ MPa (table 1). C is a dimensionless constant of order unity [36,40–42,114], which generally depends on the contact area between blade and crack tip, the friction coefficient and the strain stiffening characteristics of the cut material [36,40–42,114]. We follow Püffel *et al*. [28] and assume C≈2, so that, for pristine mandibles, Ω≈0.02–0.009, consistent with the condition Ω<0.05—the effect of tool geometry should be negligible across all pseudoleaves, as observed. The edge radii of forager mandibles, however, showed strong negative allometry ($R_{\text{f}}\propto m^{-0.87}$) and varied from a minimum of 89 nm to a maximum of 11712 nm (figure 4b). Ω can now be substantially larger: for 4 : 1 pseudoleaves, it may be as large as 1.4, consistent with the condition that Ω≈1, and leading to fracture force predictions of about 100 and 50 mN for 400 and 200 µm leaves, respectively, in excellent agreement with the measured forces (figure 2). For 10 : 1 pseudoleaves, the material length scale is larger, and the effect of wear on cutting forces is thus smaller: even for the most worn mandibles, Ω remains smaller than about 0.7, and the predicted cutting forces thus change from physical minima of about 40 and 85 mN to maxima of 70 and 140 mN for thin and thick films, respectively, again in robust agreement with the experimental data (figure 2).

Given the simplicity of the model, the qualitative and quantitative agreement between predictions and observations is encouraging, but some uncertainty of course remains. For example, a weak trend for an increase in fracture force with mandible size persists in pristine mandibles. Two explanations may be put forward to account for this increase. First, it is possible that the technically challenging radius measurements carry so much uncertainty that they failed to uncover a true underlying scaling. Indeed, there was a weak, if insignificant, trend for R to increase with size. Second, the tip radius may not be the relevant length scale [37,89,91–93]. It is clear that the mandibles of larger ants are, in fact, larger, so perhaps a different characteristic dimension comes into play and is responsible for the small increase in fracture force with size.

Although there is perhaps no other insect genus single-handedly responsible for such a large fraction of the defoliation in the Neotropics, leaf-cutter ants are unusual folivores in the sense that they neither ‘chew’ nor directly consume the plant matter they harvest. It is thus prudent to consider the extent to which insights derived from a simple model of blade cutting generalize to other biting –chewing insects. A definite answer requires experimental tests, rendered challenging because the kinematics of chewing involve two opposing mandibles and are thus harder to replicate *ex vivo*; the larger mandible surface area involved in chewing also makes the choice of the relevant tool length scale less obvious. However, neither a variation in chewing kinematics nor in mandible geometry influences the minimal energetic cost that determines the lower bound for the fracture force—the general energetic picture derived from fracture mechanics, and thus the insights it affords, remains valid [36]. Indeed, first-order mechanical models of scissor cutting, perhaps the closest simple analogue to leaf processing by chewing, yield essentially the same equations [115], and models of chip cutting, used by some wood-eating insects, also involve material toughness and mandible geometry [56], as discussed here. Further research is required to sharpen our understanding of the interaction between mandible geometry, material length scale, mandible kinematics and mechanical effort across insects with biting–chewing mouthparts.

### 4.2. From pseudoleaves to leaves

Having evaluated the predictive power of the simple model for a well-defined model material with respect to two leading hypotheses, we next turn to the more complex case of cutting heterogeneous plant materials. Do the same major physical effects dominate the cutting process? Two major difficulties stand in the way of answering this question: to obtain independent measures of $G_{c}$ and to determine the appropriate choice for $\sigma_{c}$.

It has long been recognized that cutting tests—be they in form of a guillotine [90], shearing blades [116], scissors [117] or a single razor blade [24]—may well yield the best estimate for the fracture toughness of leaves, for they restrict failure to small regions close to the blade edge, so that extrinsic toughening mechanisms are circumvented [104–106,117]. Traditional fracture assays, such as notched tensile tests and pure shear tests, will instead often return elevated toughness estimates, for they permit the crack to interact with material architecture and so result in crack arresting, crack deflection or crack bridging. Consistent with this expectation, Japanese laurel leaf lamina toughness estimated via pure shear tests was almost a factor of two larger than the estimate from cutting tests ($G_{c}=746\;\pm \;210\;\text{J}\;{\text{m}}^{-2}$ versus $G_{c}=F_{f}t^{-1}=223\;\pm \;70\;\text{J}\;{\text{m}}^{-2}$, respectively; figure 3 and table 1). This disparity stands in contrast to experiments with pseudoleaves, where the two independent estimates were in excellent agreement. Physically, the fracture force cannot be lower than $F_{f}\leq G_{c}t$, which at once suggests that tests that involve free-running cracks do not provide reliable leaf fracture toughness estimates, even for relatively homogeneous Japanese laurel leaf laminae. Although extrinsic toughening mechanisms are not well-studied in plant leaves (e.g. [67,104,118]), it appears they may be potent.

A disparity between toughness estimates from tensile and cutting tests is not universal for plant tissues: Vincent [104] reported a robust agreement for approximately isotropic parenchyma; in other cases, still, notch tests yielded lower toughness estimates than cutting assays [67]. Intriguingly, leaf toughness estimated via cutting force data from forager mandibles appears to be in closer agreement with estimates from pure shear tests ($G_{c}=746\;\pm \;210\;\text{J}\;{\text{m}}^{-2}$ versus $G_{c}=F_{f}t^{-1}=\text{580}\;\pm \;\text{180}\;\text{J}\;{\text{m}}^{-2}$, respectively; Figure 3 and table 1)—the effect of a blunt blade can evidently be just as large as the effect of extrinsic toughening arising from the deflection of free-running cracks.

The importance of blade sharpness is well recognized, but the associated discussion in the plant literature typically remains on qualitative terms (e.g. [24,119]). Scissors used in previous work had edge radii of about 2−3 µm [22,67,117,120], comparable to those of forager mandibles. It is therefore plausible that the toughness estimates thus obtained were inflated, too. More recent work has sought to circumvent this problem by using razor blades, with significantly smaller cutting-edge radii (about 300 nm) [24]. At what point is a blade sharp enough? The model studied in this work may be simple, but it has the advantage that it provides an explicit connection between blade geometry and cutting force, so permitting evaluation of conditions under which blade geometry may be neglected altogether. For thin sheet cutting, we have recently argued that a cutting tool is ideally sharp only if Ω<<1 [40]—making it sharper still will not result in a further noticeable decrease in the cutting force. This condition can be used to determine a critical radius below which tool geometry may be neglected, with the principal difficulty hidden in the non-trivial definition of the characteristic stress $\sigma_{c}$: depending on the material and the fracture process, $\sigma_{c}$ may be a yield or failure stress [40,42], a shear stress [41], an elastic modulus [36,121] or the critical strain energy density [41,121]. Using a large dataset to estimate the properties of the median tropical leaf [122] provides $\sigma_{c}=3$ N mm^−2^ and $G_{c}=0.4$ N mm^−1^, so that $\Omega \approx 2\cdot 3\;(0.4{)}^{-1}R$ mm; a blade with a radius R<10 µm would thus return a toughness estimate within 5% of the true value. However, this calculation assumes that the tensile strength is an appropriate proxy for $\sigma_{c}$ and, more crucially, that the toughness data themselves, measured dominantly via scissor cutting, were not inflated by the use of blunt blades. Both may well be incorrect. More detailed experiments are required to determine the appropriate choice for $\sigma_{c}$ for leaf laminae; a suitable route may be cutting measurements with wires of variable radii [113], combined with more detailed leaf material characterization via tensile tests.

### 4.3. Mechanical strategies of an evolutionary arms race

A significant body of work sought to determine the mechanical and structural traits of plants most effective at herbivore deterrence (e.g. [ 20,60,106,116,123]). Disentangling the effect of leaf structural and material properties on insect herbivores generally remains challenging, because they often appear to be cross-correlated [116,123]. Although some leaf traits are more strongly correlated with herbivore deterrence than others [116,123], a one-fits-all condition appears unlikely, and the relevant traits likely depend on animal size and feeding guild [116,117,123]. In fact, the existing evidence is so noisy that the leaf area density, ζ=m/A (sometimes also called leaf specific mass), may often be about as good a predictor as any other mechanical or structural trait, but with the distinct advantage that it is easy, cheap and fast to measure [20]. ζ is a combined trait: it depends on both leaf thickness (t)—a structural trait—and leaf density (ρ), a material property, ζ=ρt [124]. In line with this observation, the cutting force model put forward in this work predicts that the minimum cutting force is determined by the product between a structural and a material property: leaf thickness and toughness, respectively. Leaf toughness is likely strongly correlated with leaf density, for it is approximately proportional to the relative volume fraction of the composite cell wall and thus the crude fibre content [13,68,125]. Leaf area density may thus well be a good proxy for cutting force, $F_{f}\propto \zeta$. But is increasing the cutting force via toughening or thickening equivalent in its deterrent effect [106]?

Increasing the cutting force through changes in thickness versus toughness appears *mechanically* equivalent. However, there are good reasons to argue that this mechanical equivalence will not readily translate into an ecologically relevant equivalence of deterrence efficacy. Leaf thickness generally depends on the number of layers and length of palisade cells, the placements of veins and the width of the mesophyll, epidermis, hypodermis and indumentum [124]. Thus, doubling the thickness may double the required cutting force, but it will likely also double the material gained per cut length—the net deterrent effect, then, may well be zero (larger leaf fragments may still be harder to digest, and so require more chewing, or may delay nutrient assimilation [13,23]). Increasing leaf lamina toughness by increasing the volume fraction of the usually indigestible cell wall, in turn, would make cutting more energetically challenging and less rewarding. From the perspective of the plant, increasing ζ via a high density and thus toughness may be preferable to an increase via lamina thickness, and from the perspective of the insect, ‘sensing’ toughness as opposed to absolute cutting force may be advantageous. So can they? We are not aware of any evidence in support or defiance. Tough leaves are often described as scleromorphic [125,126]. Although there is no universally agreed-upon definition of scleromorphy, qualitative ranks provided by botanists are strongly correlated [126], suggesting that humans generally sense similar traits—but it is unclear which traits exactly. A possible experimental approach to this problem may be to observe herbivore feeding preferences on leaves with different magnitudes of Loveless’s sclerophylly index (the ratio of crude fibre to crude protein, or the ratio of cell wall to cell content [125]), which provides a measure of dilution of nutrients by the cell wall [126] and correlates well with fracture toughness measured via scissor tests [125].

Of course, scleromorphic features have evolved for reasons other than herbivore deterrence, including resistance to wilting, water and nutrient conservation, to enhance longevity or to ensure efficient photosynthesis [20,22]. Where leaf thickness or toughness is otherwise constrained, a suitable and perhaps more effective deterrent may then be to render cutting costly by inducing wear. In mammalian teeth, mild wear is sometimes seen as essential for tooth function [27,127], but in insects, it can lead to reduced development and increased mortality rates, shifts in task preferences and even induce ontogenetic adaptations in mandible morphology and relative head size [48,54,108,128–132]. So strong is the assumed impact of wear that some have speculated that frequent moulting may be a counter-adaptation to it [133,134]. Consistent with earlier work by Schofield *et al.* [48], our data show that the effect of wear on leaf-cutting force can be substantial: it can cause an elevation over the minimum force by up to a factor of five (figures 2 and 3). To achieve the same increase through a change in thickness would require substantially elevated material investment, and realizing it through an increase in toughness may result in dysfunctional leaves comprised dominantly of cell wall and crude fibre. The key question, then, is how plant leaves can go about rapidly inducing wear and how insects should best avoid it. Two well-known strategies include the incorporation of exogenous grit or silica phytoliths in plants [23,135–137] or of transition metals in insect mandibles [53,138,139]. However, direct evidence for a positive or negative effect on wear rate is rare [139] and often ambiguous if not conflicting (e.g. [54,129,132,140]). Understanding the biomechanics of mandibular wear, from the perspective both of the plants and of the insects, is a priority area for future work.

## Data Availability

The data are provided in a Dryad repository [141]. Supplementary material can be found in the online figshare repository. Electronic supplementary material is available online [142].
